# Cricothyroid Approximation Surgery (Type IV Thyroplasty): Insights From Failure Cases and an Analysis of Contributing Factors Using Three-Dimensional Computed Tomography

**DOI:** 10.7759/cureus.80310

**Published:** 2025-03-09

**Authors:** Ray Motohashi, Hiroyuki Hiramatsu, Ryoji Tokashiki, Eriko Sakurai, Kiyoaki Tsukahara

**Affiliations:** 1 Otorhinolaryngology - Head and Neck Surgery, Tokyo Medical University, Tokyo, JPN; 2 Otolaryngology, Hiramatsu Ear, Nose, and Throat (ENT) Clinic, Tokyo, JPN; 3 Otolaryngology, Shinjuku Voice Clinic, Tokyo, JPN

**Keywords:** 3d ct, cricoid cartilage, cricothyroid approximation, feminization, high pitch, phonation, thyroid cartilage, tp iv, type iv thyroplasty

## Abstract

Type IV thyroplasty (TP IV) is commonly regarded as a simple surgical procedure to raise the pitch of the speaking fundamental frequency (SFF) by lengthening the vocal folds through the approximation of the thyroid and cricoid cartilages. However, we report two cases in which TP IV was unsuccessful due to excessive shortening of the cricothyroid distance as analyzed by three-dimensional computed tomography (3D CT). One case was performed under general anesthesia and the other under local anesthesia. In normal high-pitched phonation, the rotational movement of the thyroid and cricoid cartilages and the anterior gliding movement of the inferior horn of the thyroid cartilage play essential roles in vocal fold elongation. The anterior gliding movement shifts the inferior horn, which serves as the fulcrum of rotational motion, forward to effectively elongate the vocal folds. However, when the cricothyroid distance is excessively short, the fulcrum shifts backward, preventing adequate elongation of the vocal folds. Understanding the physiological movements involved in normal high-pitched phonation is crucial for the success of TP IV. Performing the procedure while monitoring the voice under local anesthesia allows for better assessment of vocal function, with particular attention to the movement of the oblique part of the cricothyroid muscle. Additionally, excessive traction at the midline should be avoided to prevent complications.

## Introduction

Type IV thyroplasty (TP IV) [1], a cricothyroid approximation procedure, and anterior glottoplasty [2,3], a vocal cord shortening procedure, have been reported to increase the pitch of the speaking fundamental frequency (SFF). Recently, the diagnosis and treatment of gender identity disorder (GID) have been initiated in Japan, and the demand for voice surgery in male-to-female (MTF) patients has also increased. Contrastingly, the 2024 Japanese Guidelines state that voice surgery for MTF is a personal decision, similar to other cosmetic surgeries [4]. Patients should seek a specialist’s opinion and proceed with caution. Presently, pitch elevation surgery is not commonly conducted in Japan. TP IV is widely called a relatively simple surgical technique for SFF and is often performed in surgery for MTF, along with arytenoid adduction for vocal fold paralysis [5]. We encountered an unsuccessful case of Adam’s apple resection with TP IV in Thailand. Additionally, we encountered another case in which the SFF could not be raised even under local anesthesia for the same reason. In these case studies, we examined high-pitched phonation and TP IV using three-dimensional computed tomography (3D CT).

## Case presentation

Case 1

A 23-year-old man with GID underwent Adam’s apple resection and TP IV under general anesthesia in Thailand. Given that the SFF remained unchanged, the patient visited our hospital.

Endoscopic Findings at First Visit

**Figure 1:**
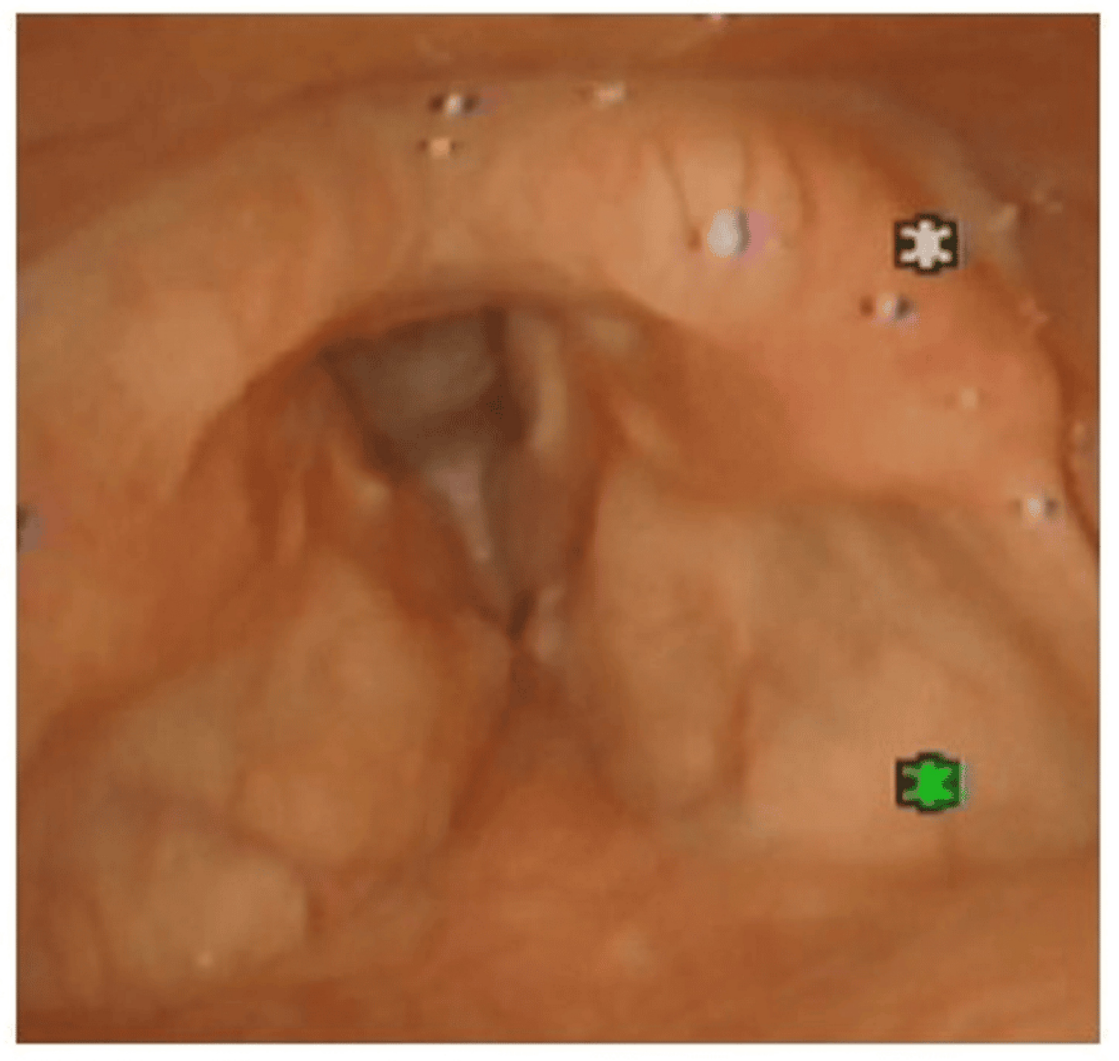
Endoscopic findings at the initial visit of Case 1. Shortening of the vocal folds was observed when viewed endoscopically, and the anterior commissure was somewhat undefined. Vocal-fold mobility did not reveal any abnormalities.

The vocal fold length was shortened, and the anterior union was slightly unclear (Figure 1). No abnormality was observed in the mobility of the vocal fold.

Voice Findings

The SFF was 116 Hz, which did not increase compared to preoperative levels.

No information on the surgeries performed in Thailand was present. A 3D CT was conducted to obtain detailed information about the larynx.

3D CT Results

**Figure 2:**
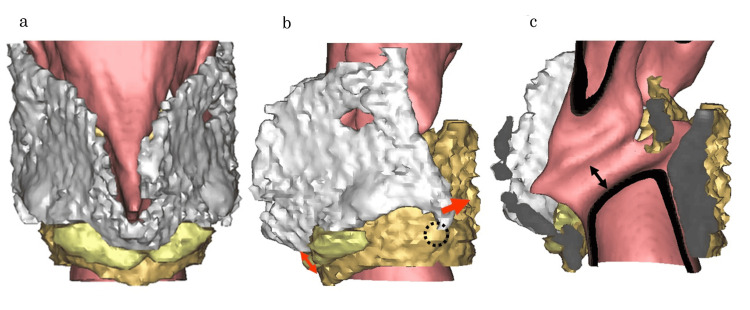
3D CT results of Case 1. (a) Frontal view. (b) Lateral view. (c) Lumen. White: thyroid cartilage; bronze: cricoid cartilage; yellow: Gore-Tex® (WL Gore and Associates, Inc., Flagstaff, US). The cricoid and thyroid cartilages were overlapped, with the cricoid cartilage entering the thyroid cartilage (red two headed arrow). The inferior horn of the thyroid cartilage was deviated considerably posterior to the cricothyroid joint (red arrow). The vocal cords had not lost thickness (black two headed arrow). 3D CT: Three-dimensional computed tomography

The cricoid and thyroid cartilages overlapped with the cricoid cartilage extending inside the thyroid cartilage. The inferior horn of the thyroid cartilage was substantially displaced posteriorly from the cricothyroid joint. The vocal fold had not thinned. Additionally, the thyroid cartilage resection extended to the anterior commissure (Figure 2).

We suspected that this was due to excessive traction on the cricoid and thyroid cartilage and attempted TP IV release under general anesthesia.

Surgical Findings

An external incision was made to note the thyrocricoid space. Both cartilage types were fixed and could not be released. The vocal cords were seen using a direct laryngoscope. The cricoid cartilage protruded below the glottis and was in contact with the vocal folds.

Case 2

A 46-year-old man with GID underwent TP IV under local anesthesia at another hospital. Given that the SFF remained unchanged postoperatively, the patient visited our hospital.

Endoscopic Findings at First Visit

The vocal fold mobility was normal, and no abnormal findings were noted.

Voice Findings

The preoperative SFF was 130 Hz. During the assessment, the SFF was 130 Hz with no change. A 3D CT was conducted to obtain detailed information about the larynx.

3D CT Results

**Figure 3:**
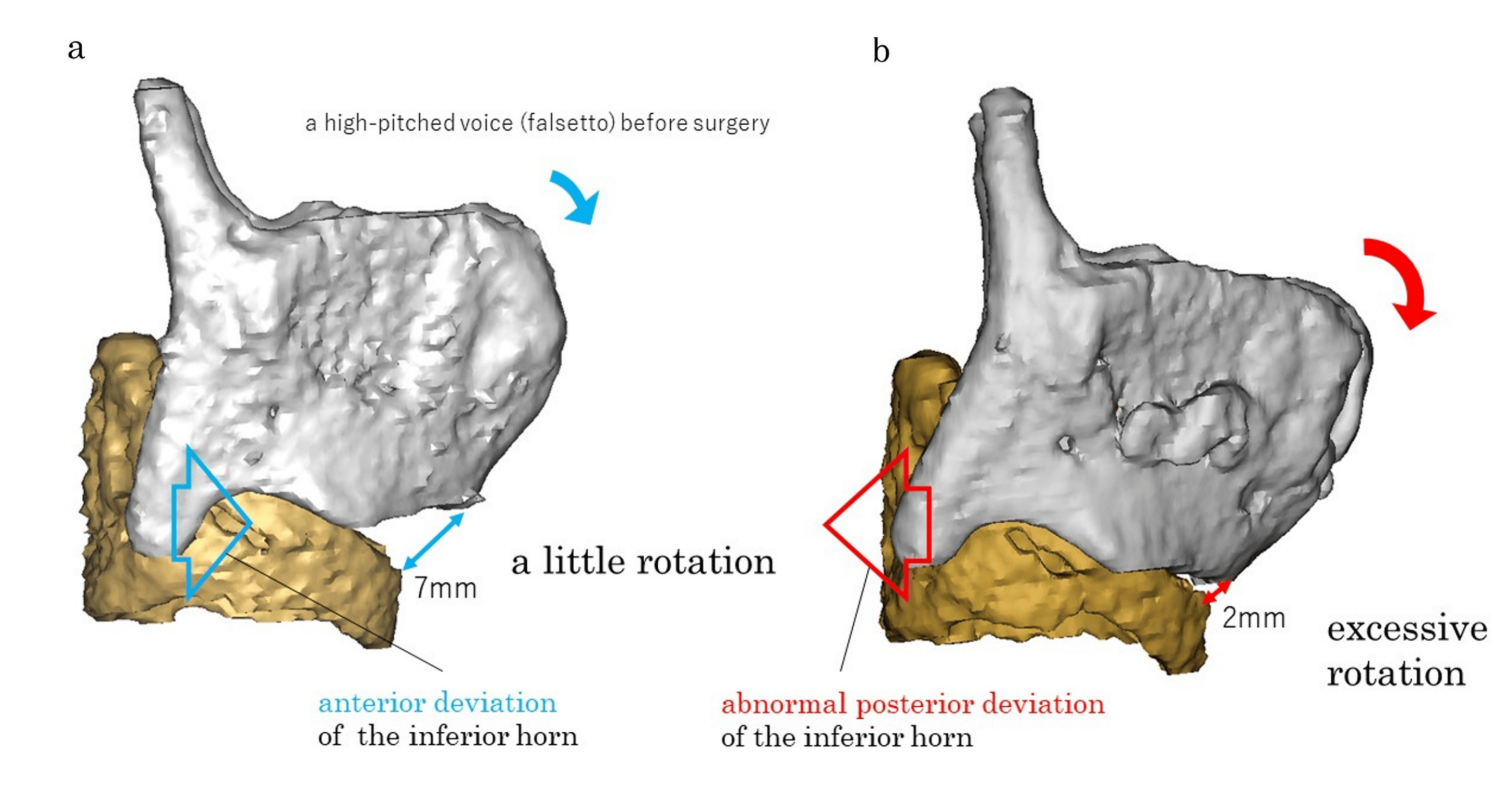
3D CT of high-pitched phonation before and after the surgery in Case 2. (a) High-pitched phonation before the surgery. (b) Excessively approximated results after type IV thyroplasty. Excessive traction can shorten the distance between the cricoid and thyroid cartilages. This may lead to the inferior horn of the thyroid cartilage deviating posteriorly instead of anteriorly such that the vocal folds cannot be extended. 3D CT: Three-dimensional computed tomography

**Figure 4:**
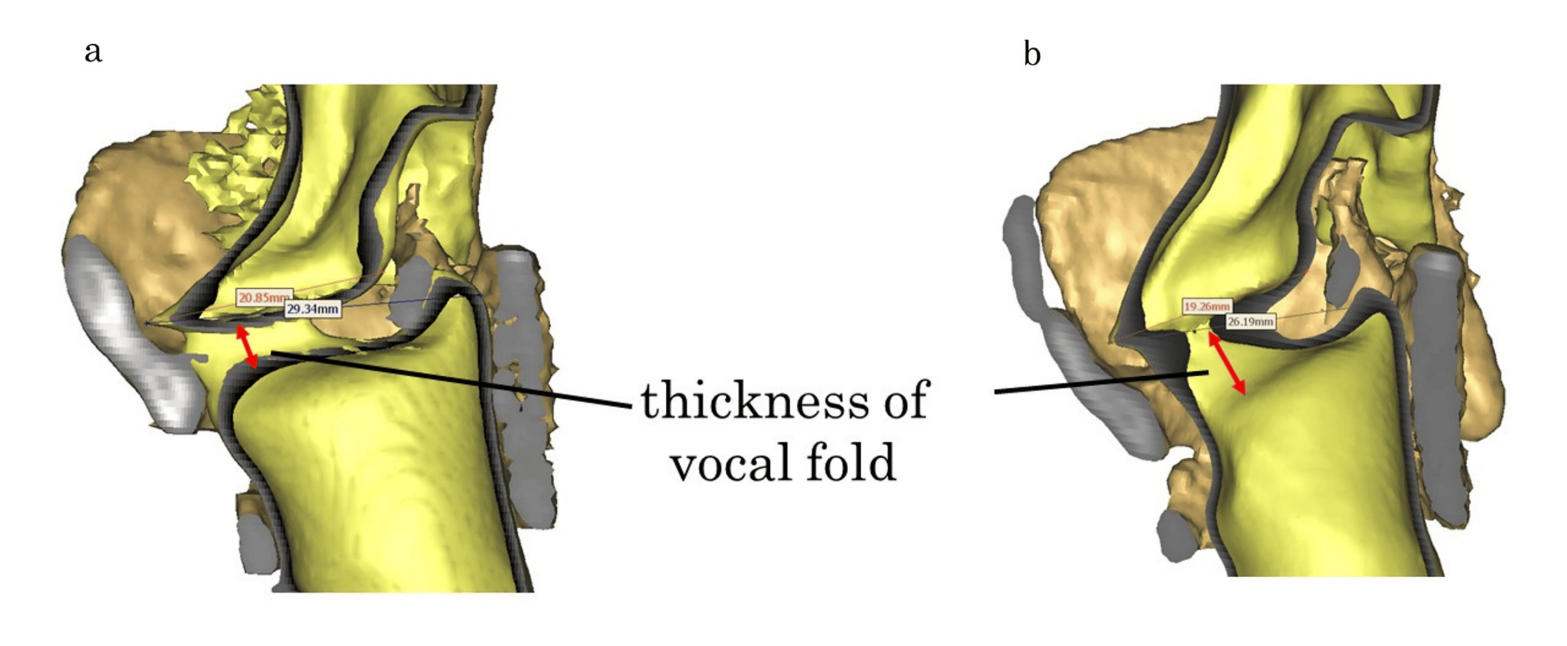
3D CT (lumen) of high-pitched phonation before and after the surgery in Case 2. (a) High-pitched phonation before the surgery. (b) Excessively approximated results after type IV thyroplasty. The length of the vocal folds was shortened, and the thickness of the vocal folds was increased (red two headed arrows). No change in SFF was noted (Preoperative 130Hz, Postoperative 130Hz). 3D CT: Three-dimensional computed tomography; SFF: Speaking fundamental frequency

Preoperative CT data were obtained so that preoperative and postoperative comparisons could be made. Postoperatively, the cricoid and thyroid cartilages were close to each other. The inferior horn of the thyroid cartilage was posteriorly displaced. The vocal fold length was shortened, and the vocal fold thickness was increased (Figures 3-4).

We speculated that excessive traction might have caused this phenomenon, even though the surgery was performed under local anesthesia and while listening to the patient's voice.

## Discussion

**Figure 5:**
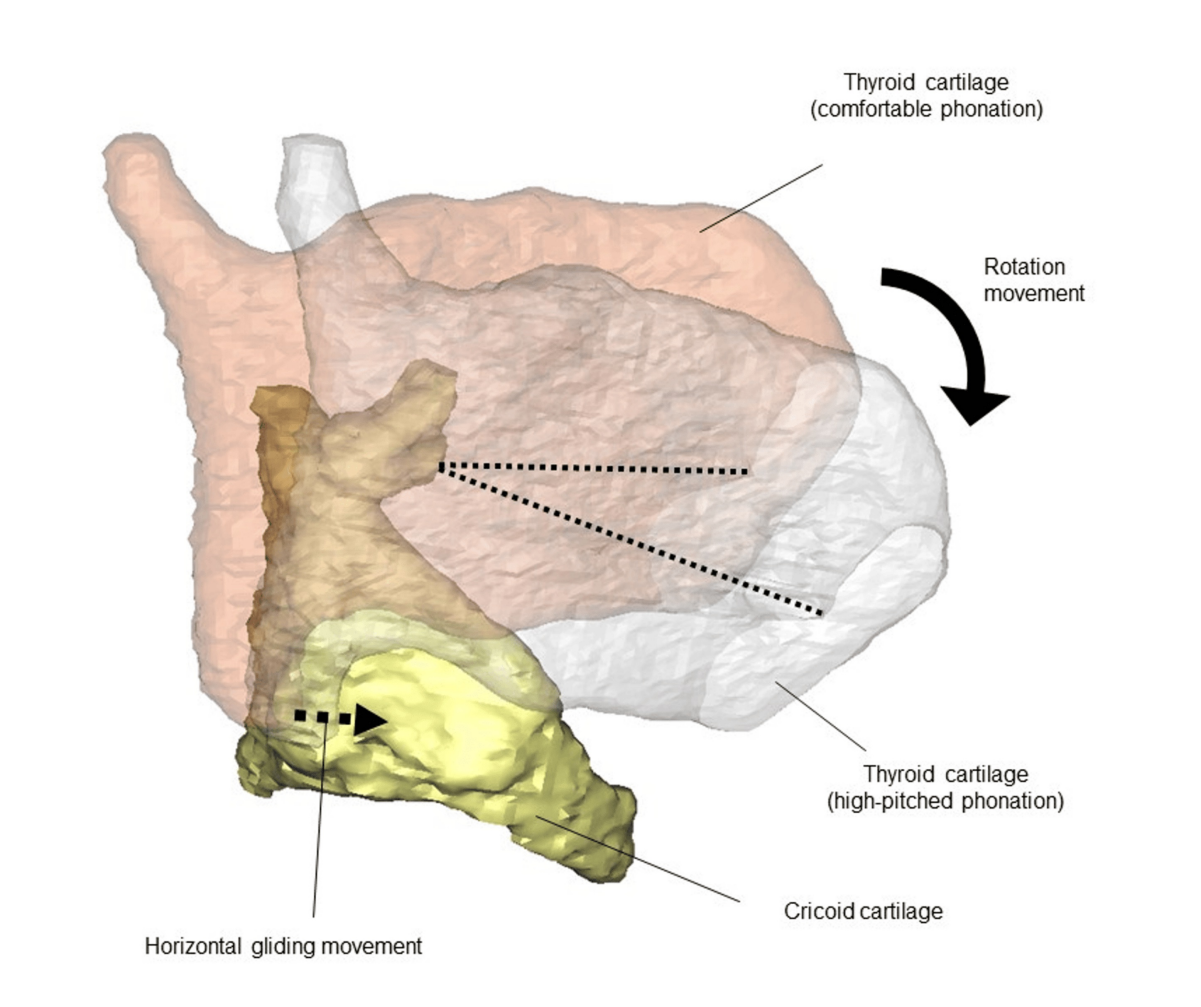
3D CT of normal and high-pitched phonation. Dotted lines: Length of the anterior commissure from the base of the vocal fold process. Rotational movement involves the straight part of the cricothyroid muscle, whereas gliding movement is performed by the oblique part of the cricothyroid muscle. 3D CT: Three-dimensional computed tomography The figure is adapted from Hiramatsu et al. [12], with permission obtained for its use.

TP IV was first reported by Isshiki in 1974 [1], and it is widely recognized as a simple surgical procedure for increasing the pitch of the SFF. The vocal folds can be lengthened, and higher pitched speech can be achieved by approximating the thyroid and cricoid cartilages. Research on cadaver dissections has revealed that high-pitched phonation can be achieved via rotational and gliding movements through the straight and oblique parts of the cricothyroid muscle [6,7]. Our institution has previously reported on the usefulness of 3D CT for understanding the three-dimensional configuration of the laryngeal framework [8-11]. In a live patient study using 3D CT, Hiramatsu et al. [12] demonstrated that straight and oblique sections simultaneously work to produce efficient, high-pitched phonation (Figure 5).

The TP IV aims to reproduce the motion of the straight part of the cricothyroid muscle. However, excessive traction posteriorly displaces the inferior angle. The inferior horn, the fulcrum of rotational movement by the straight part of the cricothyroid muscle, moves posteriorly, resulting in inefficient extension of the vocal folds and postoperative challenges with high-pitched speech in some cases.

Case 1 was conducted under general anesthesia, and voice monitoring was not performed. This is based on the general theory that cricothyroid closure is equivalent to vocal fold stretching. However, excessive proximity between the cricothyroid spaces caused a large displacement of the inferior horn posteriorly, and vocal fold extension was not achieved. Kurita et al. [13] did not refer to the inferior horn and reported a case of cricothyroid cartilage invaginated into the thyroid cartilage due to excessive proximity between the cricothyroid cartilage in TP IV under general anesthesia in Southeast Asia. In this case, the cricoid and thyroid cartilages were in contact with each other, causing irreversible fixation. Mesuda et al. [14] reported voice disorders following pitch elevation surgery in Southeast Asia, although the details are unavailable. The authors pointed out that few established surgical procedures exist for pitch elevation, which include some technical issues. However, Haji and Tate [15] reported that TP IV is a simple and reversible procedure that can be performed under local anesthesia and has become one of the most common procedures used recently for pitch elevation surgery [16]. Additionally, this procedure allows for the simultaneous removal of the Adam’s apple and is often used to feminize the voice [17,18].

In some cases, shortening of the cricothyroid distance via excessive traction can displace the inferior horn of the thyroid cartilage posteriorly rather than anteriorly, making it impossible for vocal fold elongation. Excessive Adam’s apple removal should be carefully performed because of the additional risk of entry into the laryngeal lumen and voice changes due to anterior commissure disruption. Surgeons should be aware of these potential complications when performing this procedure [19,20]. In Case 2, the cricoid and thyroid cartilages were too close together, and the inferior horn shifted backward, shortening the vocal cord length and increasing the vocal fold thickness (Figure 4). This operation, performed under local anesthesia, was likely affected by excessive traction due to the mistaken belief that reducing the cricothyroid distance would directly lead to greater vocal fold elongation, resulting in excessive approximation of the cricoid cartilage and the inferior margin of the thyroid cartilage.

Hiramatsu et al. [12] reported that the inferior horn moved forward in all normal cases when high-pitched phonation was performed. Additionally, they reported an average shortening of the cricothyroid distance of 2.40 ± 1.88 mm when high-pitched phonation was conducted from normal phonation. In other words, even mild rotational movements are sufficient for high-pitched speech production if the inferior horn sufficiently moves forward. In TP IV, the gliding movement of the oblique part of the cricothyroid muscle is crucial. This surgery must be performed under local anesthesia with voice monitoring and should not be performed under general anesthesia. In addition, even with local anesthesia, excessive traction in the midline must be avoided.

As these were rare cases, we were unable to conduct detailed subgroup analyses or draw definitive conclusions. Future research studies should aim to analyze larger sample sizes to verify our findings. Another limitation is the limited reporting of failed cases, which poses challenges in establishing a comprehensive comparative framework. The lack of detailed accounts of unsuccessful outcomes restricts the contextualization of findings within the broader literature. Addressing this reporting gap would contribute to a more balanced and thorough understanding of the topic.

## Conclusions

Understanding the physiological movements involved in normal high-pitched phonation is crucial for the success of TP IV. Excessive traction can cause a shortening of the distance between the cricoid and thyroid cartilages, which may result in the inferior horn of the thyroid cartilage deviating posteriorly instead of anteriorly, preventing the elongation of the vocal folds. Performing the procedure while monitoring the voice under local anesthesia allows for better assessment of vocal function, with particular attention to the movement of the oblique part of the cricothyroid muscle. Therefore, TP IV should not be performed under general anesthesia and excessive traction at the midline must be avoided to prevent complications.
